# Chromic Acid in Affections of the Tongue

**Published:** 1884-07

**Authors:** 


ARTICLE VII.

### CHROMIC ACID IN AFFECTIONS OF THE TONGUE.

Mr. Henry T. Butlin, F. R. S., has used chromic acid in certain affections of the tongue, with markedly good effect. In June, 1881, he treated two cases of glossits with a ten-grain solution of chromic acid in water, painted on the sore areas of the tongue three or four times a day. Both cases improved. A case of secondary syphilitic, deep and jagged ulcers of the tongue, and ulcerations of the inside of the cheek, which showed no improvements under hyd. c. cret., iodide of potass. or liq. bichlor., were, after a week's treatment with chromic acid solution, almost completely healed. Another case of flat mucus tubercles, due to secondary syphilis on the right border of the tongue, which had resised treatment with hyd. c. creta for about three and a half months, was almost completely cured in three weeks.

Mr. Butlin has used chromic acid in several different inflammatory conditions of the tongue, in many cases with cases with most gratifying success. In 27 cases, 20 have been cured or greatly relieved, seven having received little or no benefit. The seven cases were either of chronic superficial glossits, or of *tertiary* syphilis. The twenty include seven of chronic superficial glossitis, and thirteen of various *secondary* syphilitic affections. Mr. B. concludes that chromic acid cures with marvelous rapidity *secondary* affections, ulcers, mucous tubercules, and condylomata. It produces no appreciable effect on *tertiary* affections, gummata, extensive ulcers, or tubercular syphilides. Some cases of chronic superficial glossitis, with slight ulceration and renewed inflammation, are rapidly benefited by it. In cases glossitis in which the tongue surface is attacked by a fresh inflammation of great severity, glycerite of boracid acid and soothing remedies are more suitable, chromic acid rendering these worse. He reports one case of *tertiary* syphilitic ulcers of the tongue which was cured in about two months by combined chromic acid and mercury treatment, although it had obstinately resisted purely anti-syphilitic treatment for many months. The strength of the solution usually employed is grs. x–Drams j water; in some cases grs. xv–Drams j. The patient is told to paint the diseased parts three or four times a day with a camel's-hair brush dipped in the solution. There is seldom any pain or discomfort; sometimes a little smarting at first.—*Med. News.*

---

